# An eco-friendly synthesized mesoporous-silica particle combined with WSe_2_-graphene-TiO_2_ by self-assembled method for photocatalytic dye decomposition and hydrogen production

**DOI:** 10.1038/s41598-018-31188-w

**Published:** 2018-08-24

**Authors:** Lei Zhu, Dinh Cung Tien Nguyen, Jung-Hun Woo, Qinfang Zhang, Kwang Youn Cho, Won-Chun Oh

**Affiliations:** 1 Key Laboratory for Advanced Technology in Environmental Protection of Jiangsu Province, Yancheng Institute of Technology, Yancheng, 224051 China; 2 Department of Advanced Materials Science & Engineering, Hanseo University, Seosan, 31962 Korea; 3 Korea Institutes of Ceramic Engineering and Technology, Soho-ro, Jinju-Si, Gyeongsangnam-do Republic of Korea

## Abstract

To address the limitations of titanium dioxide (TiO_2_) and expand the applicability of the photocatalytic activity of TiO_2_,WSe_2_ and silica, an eco-friendly, self-assembled method for combining a silica precursor with a WSe_2_-graphene-TiO_2_ composite with cetyltrimethylammonium bromide (CTAB) as surface active agents is proposed. Firstly, for the main target, the photocatalytic degradation of organic dye solutions with different initial pH levels and catalyst dosages under visible light irradiation was surveyed. The as-synthesized sample exhibited highly efficient photocatalytic effects for the treatment of the SO dye solution in the optimal conditions of this study, which included a solution with a pH level of 11 and 0.05-gram dosage of the catalyst. Secondly, previous photocatalytic hydrogen production studies reported markedly better outcomes with SiO_2_/WSe_2_-graphene-TiO_2_ than with the binary WSe_2_-graphene and ternary WSe_2_-graphene-TiO_2_ composites under ambient conditions with and without 20% methanol sacrificing reagents. The SiO_2_/WSe_2_-graphene-TiO_2_ composite is promising to become a potential candidate for photocatalytic performance that performs excellently as well as offer an efficient heterosystem for hydrogen production.

## Introduction

Enhanced photocatalytic activity has attracted much attention from many scientists around the world due to its ability to address water pollution. Environmental pollution, which is considered a consequence of increasingly uncontrolled industrialization, has severely impaired human health and many other living organisms, as well as caused irreversible natural damage^1,2^. Among them, the effect of excess dyes and pigments in aquatic systems is considered to be the main cause of water pollution that negatively effects all aqua species due to the large volume of toxic ingredients^3^. At the same time, the renewable energy field is increasingly receiving more attention due to its great potential^4,5^, which includes the production of hydrogen by semiconducting photocatalytic materials that cause an active water splitting reaction to produce hydrogen under light irradiation^6–8^. One type includes water splitting over graphene-based composites. The invention of graphene is considered an important step forward for the field of materials science due to the outstanding benefits it offers^9^.

The coupling of graphene with a photocatalyst can enhance photocatalytic activity and fulfill practical requirements. With the current aggregation of individual graphene sheets and nanomaterials, the graphene surface interacts with numerous inorganic materials to improve the photocatalytic performance of organic dyes^10–13^. Similarly, due to their photocatalytic activities, a semiconductor with a two-dimensional (2D) TMDCs (transition metal dichalcogenides) source was investigated to help overcome some of the limitations of most materials such as only displaying photocatalytic properties when activated with ultraviolet radiation (for a large band gap materials case) or exhibiting a fast recombination phenomenon (for a small band gap semiconductor case)^14–17^.

Among the TMDCs family, tungsten diselenide (WSe_2_) is a semiconductor with a suitable band gap of approximately 1.2 eV for bulk which is a potential photocatalyst under visible light^18–20^. As mentioned previously, due to the fast electron-hole recombination, WSe_2_ has not yet led to a breakthrough for photocatalytic performance^21^. Thus, the combination of WSe_2_, graphene nanosheets and other suitable catalysis candidates can likely enhance photocatalytic properties. Ruishen Meng *et al*. reported the design of graphene-like gallium nitride and WS_2_/WSe_2_ nanocomposites for photocatalyst applications. The results demonstrated that the photocatalyst performance improved due to the prevention of the electron-hole pairs from recombination as well as the suitability of the two heterostructures’ band alignment^22^. Bo Yu *et al*. presented the enhanced photocatalytic properties of graphene modified few-layered WSe_2_ nanosheets^16^. Furthermore, many reports have presented on the combination of graphene oxide and TiO_2_. Therefore, WSe_2_, with its narrow band gap, was selected for pairing with TiO_2_ to address the limitations of TiO_2_ and expand the applicability of the photocatalytic activity of both TiO_2_ and WSe_2_.

For some time, photocatalysts that are supported by materials with large pore sizes and high porosity has attracted keen interest^23^. Due to its great advantages such as a large, specific surface area, large pore volume, and uniform and adjustable nano pore size, mesoporous silica is a promising material source for photocatalytic activity^24,25^. Furthermore, mesoporous materials, such as mesoporous silica, have been used as supports for different metal oxide nanoparticles that enhance the catalytic performances over their non-supported analogues^26–29^. Xavier Collard *et al*. synthesized the novel mesoporous ZnO/SiO_2_ composites for the photodegradation of organic dyes. Materials with a mesoporous structure that possessed a high surface area and a narrow pore size distribution exhibited the effective photodegradation of rhodamine B with good results^30^.

In this work, a SiO_2_/WSe_2_-graphene-TiO_2_ nanocomposite was synthesized using a simple method by using the silica precursor (tetraethyl orthosilicate-TEOS) at a pH level of 9.5–10, with cetyltrimethylammonium bromide (CTAB) as surface-active agents. The as-obtained composites were characterized via XRD, nitrogen adsorption/desorption isotherms, SEM, TEM, SAED, Raman spectroscopy, UV-vis DRS, XPS and PL. Furthermore, the photodegradation experiments under visible light irradiation were then proceeded with aqueous solutions of organic dyes with different initial pH levels and catalyst dosages. The recycling experiments were surveyed to investigate the photocatalyst stability. The photocatalytic hydrogen production studies of the as-synthesized nanocomposites were tested with an aqueous solution containing 20% methanol as the sacrificial reagent.

## Results and Discussion

### Characterization

**Figure 1:**
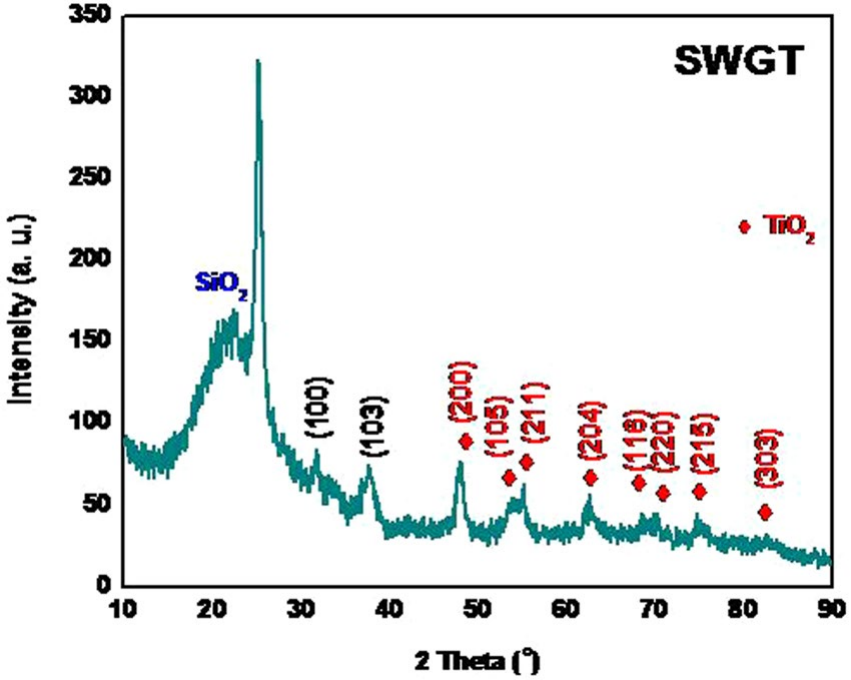
XRD patterns of the SiO_2_/WSe_2_-graphene-TiO_2_ (SWGT) composite.

XRD pattern of the SWGT composite was identified by the SiO_2_, WSe_2_ and TiO_2_ signals, as shown in Fig. 1. First, the presence of silica occurred through a broad diffraction peak at 2θ of 23.0° due to the dominant effect of the silica^31–34^. Next, the hexagonal phase of WSe_2_ can be prove by (100) and (103) planes (JCPDS No. 38–1388)^35^. Furthermore, the crystalline phase of TiO_2_ was indexed to the anatase structure, corresponded to (200), (105), (211), (204), (116), (220), (215) and (303)^36–38^. The signals of these single peaks in the SWGT composite provided evidence of the development of SiO_2_, WSe_2_, and TiO_2_ on the surface of naked graphene nanosheets that were slightly impure.

**Figure 2:**
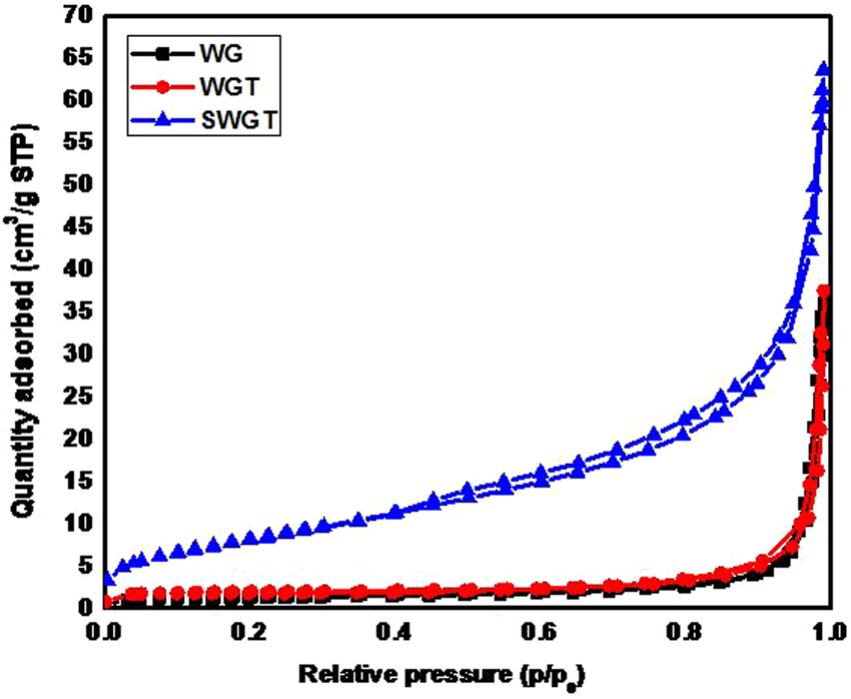
Nitrogen adsorption/desorption isotherms for the WG, WGT, and SWGT composites.

According to the results in Fig. 2, WG, WGT and SWGT composites exhibited a similar type-II curve. Results of BET surface area analysis technique and the pore size and total pore volume, which were obtained from nitrogen adsorption/desorption isotherms of the different survey composites (see Table 1). According to the BET method, the surface area was about 4.87 and 7.14 m^2^/g for the WG and WGT composites, respectively. Simultaneously, the surface area of the SWGT was calculated as 30.67 m^2^/g, which was the highest specific surface area among all the survey samples as well as more than 5 and 7 times that of the WG and WGT composites, respectively. In parallel with the highest specific surface area, SWGT also exhibited a much stronger structure with the highest total pore volume of 7.05 cm^3^/g. The total pore volumes of WG and WGT composites were 1.12, and 1.64 cm^3^/g, respectively, which were about 7 times less than that of the SWGT composite. Moreover, there was a big difference in how much the WG, WGT and SWGT composites were inhibited by the average pore size values (BJH method) which were 76.49, 103.54, and 43.57 nm, respectively.

**Table 1:** BET surface area analysis technique and the pore size and total pore volume of WG, WGT and SWGT composites.

Sample	BET (m^2^/g)	Total pore volume (cm^3^ g^−1^)	Average pore size (nm)
WG	4.87	1.12	76.49
WGT	7.14	1.64	103.54
SWGT	30.67	7.05	43.57

**Figure 3:**
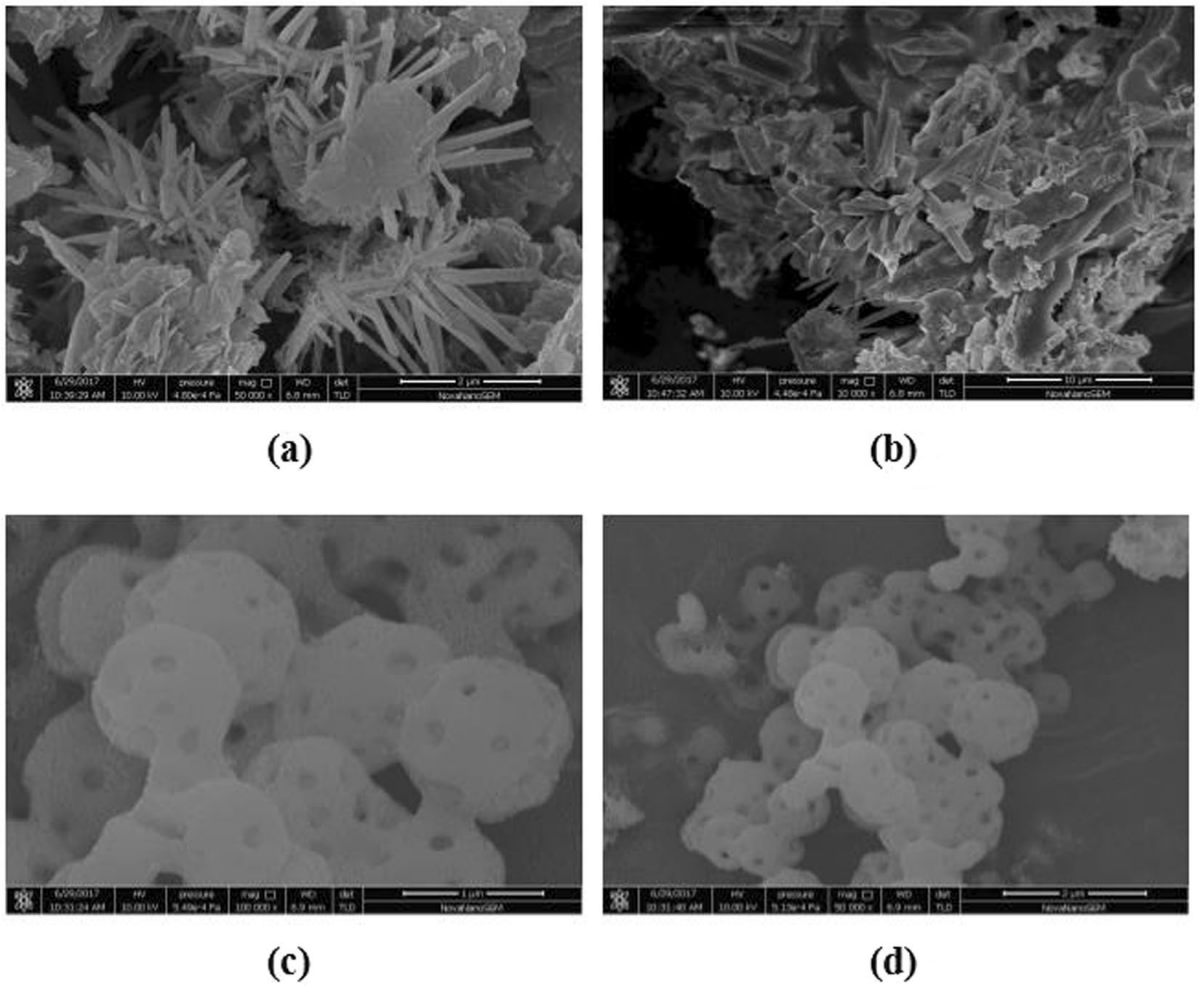
SEM morphology of (**a**) WG, (**b**) WGT and (**c**,**d**) SWGT nanocomposites.

Figure 3 provides the typical images as well as the shape and structure of the survey samples, which were analyzed using the SEM method. As SEM results of the WG composite in Fig. 3(a) suggest, the fine WSe_2_ particles existed with a rod-like morphology that was approximately 1–10 µm in length. The fine WSe_2_ particles still exhibited the rod-like morphology well in the WGT composite near the presence of the irregular-shaped anatase TiO_2_ particles, as shown in Fig. 3(b). As expected, the as-prepared SWGT exhibited the SiO_2_ nanoparticles with small-sized spherical shapes and good particle dispersion, as seen in Fig. 3(c,d). More importantly, the highlight was the presence of the micro porous holes on the surface of the round SiO_2_. This phenomenon indicated increased photocatalytic activity with the SWGT composite due to their high contact area with the pollutant organic dyes.

**Figure 4:**
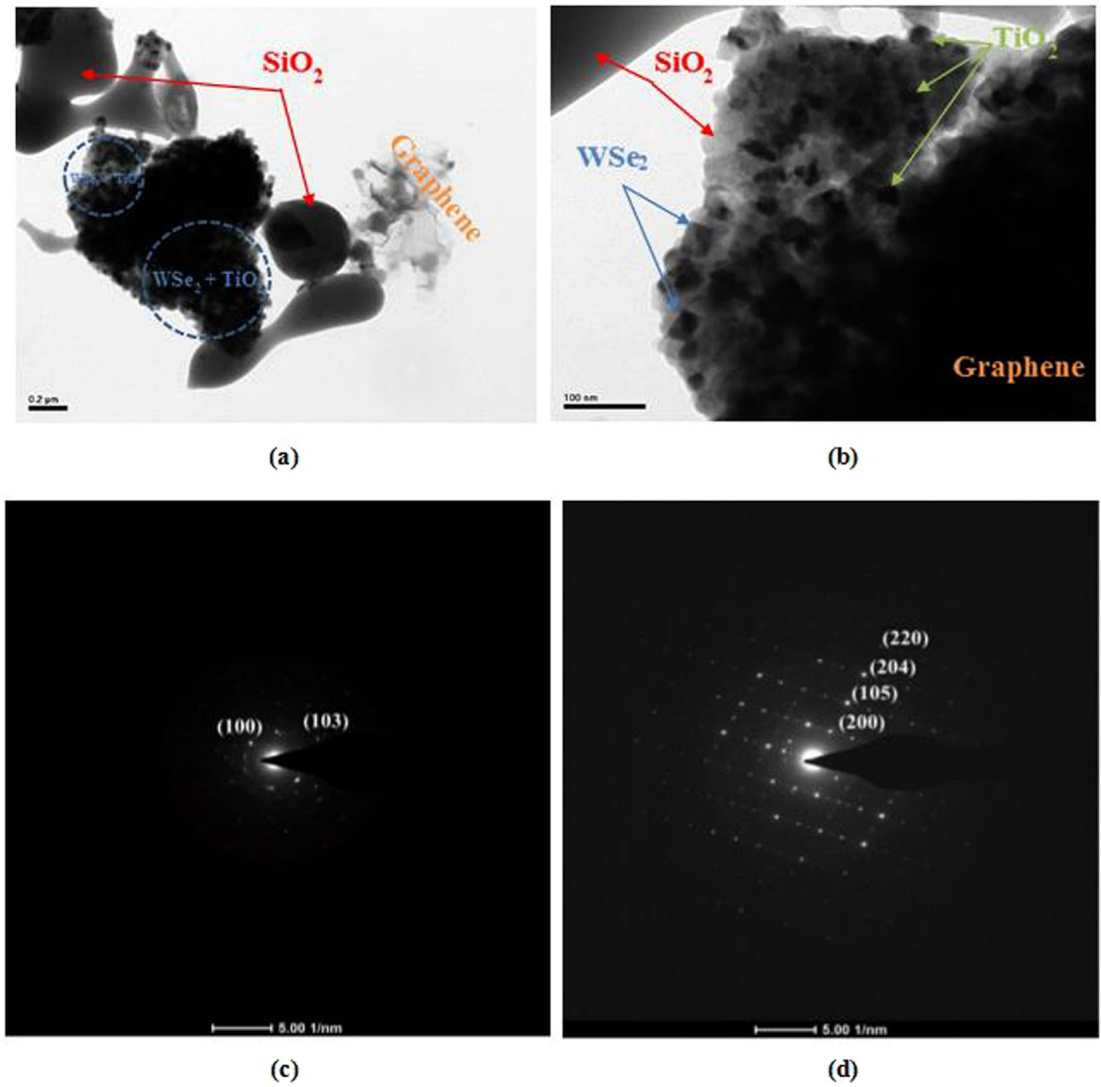
TEM images (**a**,**b**) and SAED patterns (**c**,**d**) of the SWGT composite.

TEM images were recorded, and the results are provided in Fig. 4. According to the TEM images, the typical morphologies of SiO_2_, WSe_2_, TiO_2_, and graphene were confirmed. As displayed in Fig. 4(a), a combination of WSe_2_ and TiO_2_ nanoparticles, were darker in color and decorated onto the surface of the silica. Moreover, the graphene layers clearly presented as transparent nanosheets (see Fig. 4(a)). To collect additional information, the WSe_2_ nanoparticles were exhibited as rectangle shape blocks, wherein the diameter of the particles was observed to be in the range of (25–36) nm. Specifically, we observed some spherical TiO_2_ particles of uniform sizes in the nanocomposite (see Fig. 4(b)), which formed a mixture with the rectangle-shaped blocks of WSe_2_ and covered the surface of the silica and the graphene sheets. From the achieved SAED patterns in Fig. 4(c), the d spacing values of 0.28, and 0.25 nm attributed to the Debye-Sherrer rings of (100) and (103) planes of WSe_2_ can be obtained^39^. On the other aspect, the single-phase anatase phase of TiO_2_ can be obtained on SAED pattern in Fig. 4(d), presenting the d spacing values corresponding to the (200), (105), (204), and (220) lattice planes^40^. The silica nanoparticles that were displayed as gray layers provided a large plate structure for the anchoring of the WSe_2_ and TiO_2_ nanoparticles.

**Figure 5:**
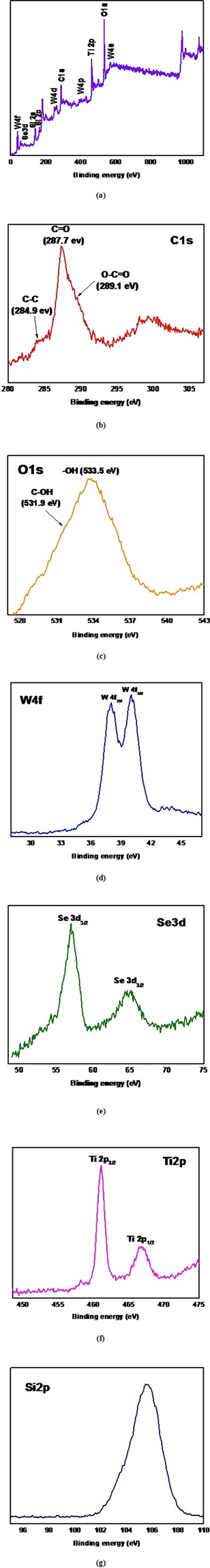
Survey XPS spectra (**a)** and high-resolution XPS spectra of C 1 s (**b**) O 1 s (**c**) W 4f (**d**) Se 3d (**e**) Ti 2p (**f**) and Si 2p (**g**) of the SWGT composite.

According to XPS analysis in Fig. 5(a), the surface bonding state of SWGT composite demonstrated the presence of W, Se, Si, C, Ti, and O elements. Following Fig. 5(b), the existence of the C-C (284.9 eV, aromatic rings), the C=O (287.7 eV) and O-C=O (289.1 eV) can be seen^41,42^. Furthermore, the peak at (531.9 and 533.5) eV was assigned to the absorbed oxygen which provided the presence of the O 1 s signal after a self-assembled reaction, as shown in Fig. 5(c). Moreover, the binding energy located at (37.9 and 40.0) eV that were ascribed to W 4f as displayed in Fig. 5(d)^43^. The presence of Se 3d, Ti 2p, and Si 2p was also identified in the XPS results, as shown in Fig. 5(e–g). The Se 3d_5/2_ and Se 3d_3/2_ binding energy peaks at (56.9 and 64.8) eV express the elemental chemical binding state of Se, while the peak positioned at (461.1 and 466.8) eV is attributed to the core level of Ti 2p_3/2_ and Ti 2p_1/2_ from TiO_2_ precursor^44,45^. XPS results in Fig. 5(g) presented the elemental chemical binding state of Si with the binding energy peaks around 105.5 eV^46^. It is worth mentioning that the above observations confirmed the successful synthesis of the SWGT composite with high impurity by a self-assembled method under basic conditions.

**Figure 6:**
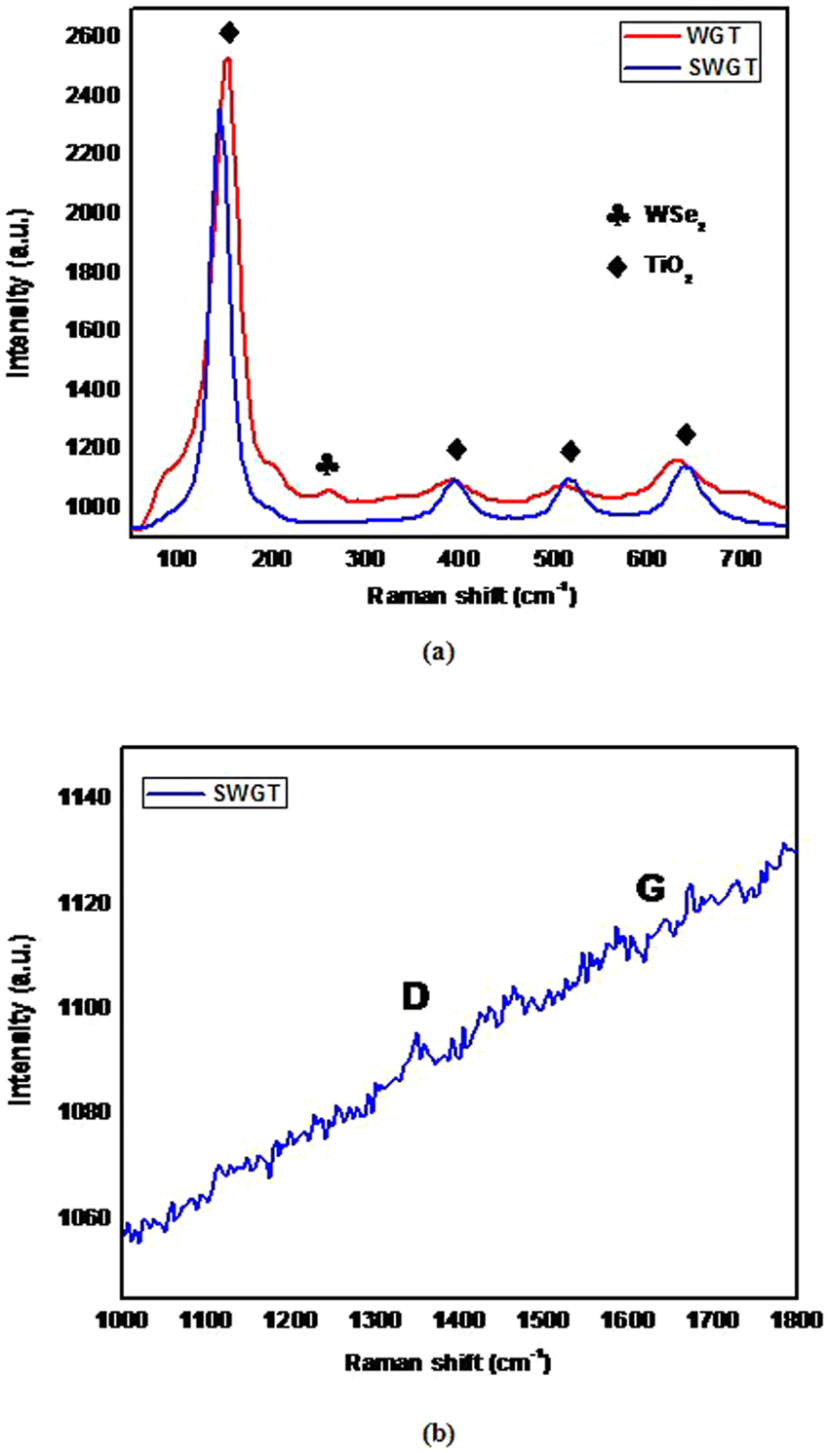
Raman spectra of the WGT, and SWGT composites.

The Raman spectroscopy of the SWGT composite is depicted in Fig. 6. The characteristic peaks of the WSe_2_ and TiO_2_ were achieved on the Raman spectra, as seen in Fig. 6(a). The obtained TiO_2_ signals were strong and displayed high intensity and located at the range shift around (150, 392, 516, and 638) cm^−1^ which were related to the E_g_(1), B_1g_(1), A_1g_ + B_1g_(2), and E_g_(2) modes of anatase TiO_2_ and confirmed the presence of TiO_2_ on the last obtained composite^47–49^. A difference in the location of the TiO_2_ peaks of the WGT and SWGT composites are provided in Fig. 6(a). After the calcination temperature condition (550 °C), the main peak at ~150 cm^−1^ of the TiO_2_ in the SWGT composite was blue shifted when compared to the WGT composite. Due to the chemical method for preparing the SWGT composite and calcination treatment, there may be oxygen defects and phonon confinement, which can lead to this kind of frequency shift along with size effect^50^. The results demonstrate that as-synthesized SWGT was successfully synthesized after a self-assembled reaction of the WGT composite with TEOS. The WSe_2_ signal was obtained in the region of 247 cm^−1^ (E^1^_2g_ band) which presented the signal of a single-layer WSe_2_^51^. Due to the dominant effect of silica, the low characteristic WSe_2_ peak overlapped. Therefore, we did not achieve the WSe_2_ signal in the SWGT Raman spectroscopy result. This outcome can be explained by the small initial amount of WSe_2_ source in the composite. According to Fig. 6(b), the existence of graphene in the SWGT composite can be confirmed by D and G bands. In the current case, D and G bands were located at (1350–1640) cm^−1^, respectively. Specifically, in our survey SWGT composite, the existence of SiO_2_ nanoparticles on the graphene surface can be led to disturb the low-intensity peak of the D-and G-band signals^52–54^.

**Figure 7:**
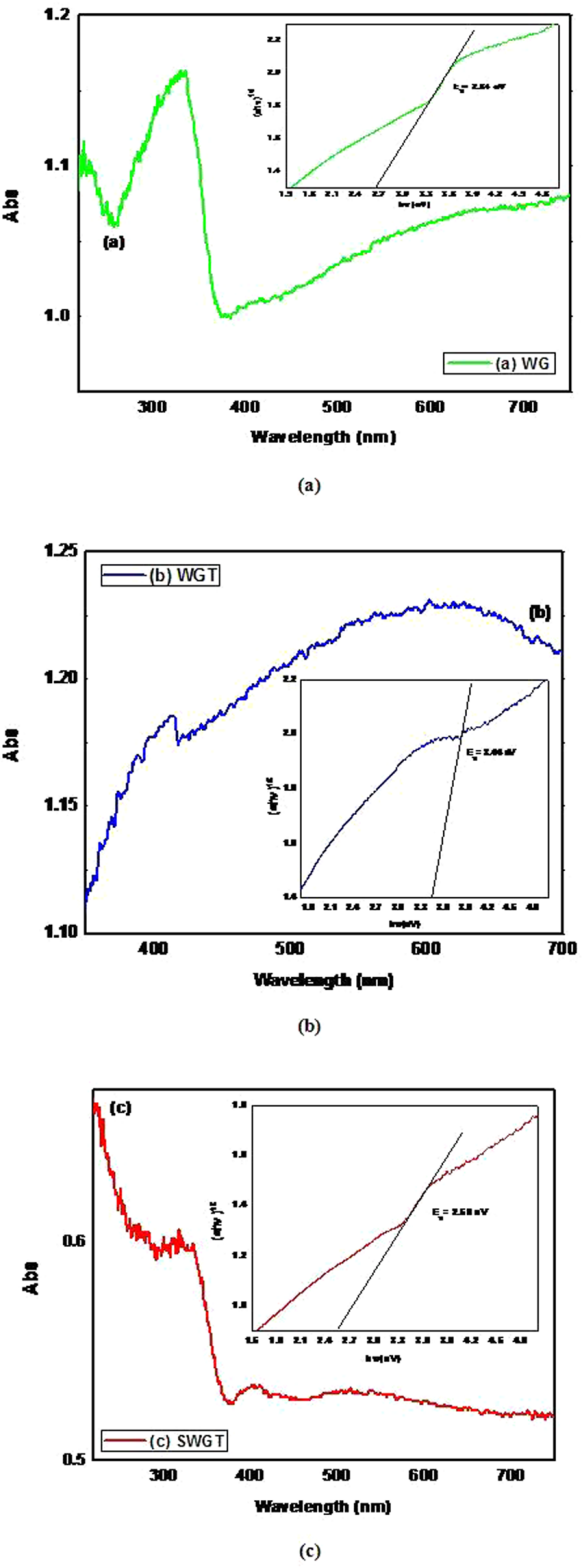
UV-vis diffuse reflectance spectrum and hν versus [(k/s)hν]^2^ graph of WG (**a**) WGT (**b**) and SWGT (**c**) composites.

The UV-vis diffuse reflectance spectrum of the WG, WGT, and SWGT composites is provided in Fig. 7. The first observation results were a maximum absorption wavelength in the visible region within a range of (400–500) nm, which corresponded to the shift of electrons from the conduction to the valence band. Following the results in Figs. 7(a–c), the absorption edge of the SWGT composite shifted to a higher wavelength than the WG composite, and red-shift absorption occurred. This phenomenon leads to improve the photocatalytic performance of SWGT composite for not only the photodegradation activity but also photocatalytic hydrogen evolution. The band gap energy that was obtained from the Kubelka-Munk transformation from the UV-vis diffuse reflection data is displayed as an insert picture in Figs. 7(a–c). The calculated band gap energies were around (2.8–3.1) eV; the samples of WG, WGT, and SWGT were (2.64, 3.44, and 2.56) eV, respectively. These results suggested that the band gap energy values of the SWGT composite corresponded to 2.56 eV, which is lower than that of both the WG and WGT composites.

**Figure 8:**
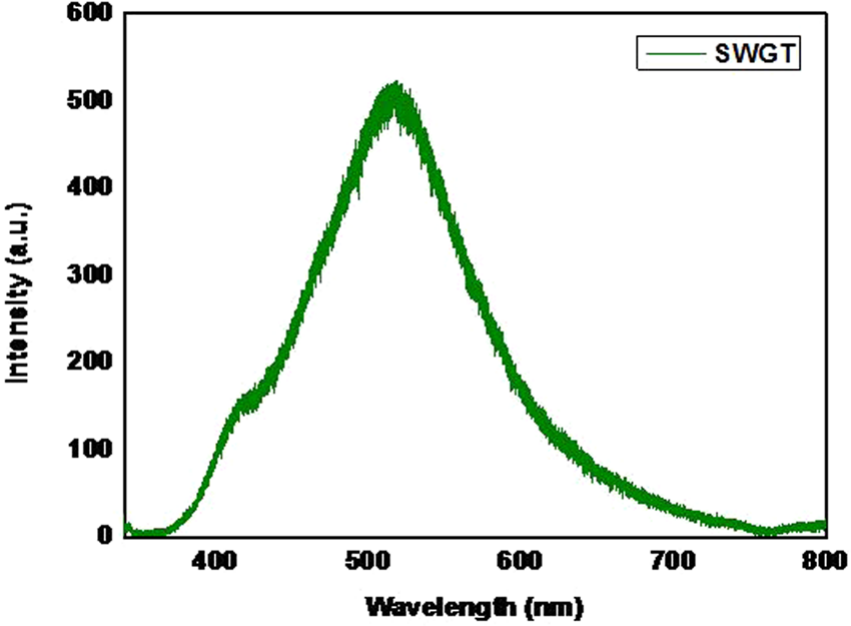
PL spectra of the SWGT composite.

PL spectra of SWGT composite with an excitation wavelength of 325 nm is presented in Fig. 8. According to the PL result, SWGT material showed the wide PL signal and a strong peak at the range of 490 to 550 nm which are related to excitonic PL mainly obtained from surface oxygen vacancies and defects of the semiconductor nanoparticles^55^. The achieved SWGT composite exhibited a greatly influenced by the intensity and response range of PL signals compared to TiO_2_-graphene, ternary photocatalyst and binary photocatalyst in other publications^50,56–58^. With the enhancing PL peak location as well as the low PL intensity, the reduced electron-hole recombination in the photocatalytic activity under visible light irradiation can be achieved and leads to enhance in the catalytic ability of the SWGT photocatalyst^59^.

### Photodegradation

#### Survey the effect of different organic dyes

**Figure 9:**
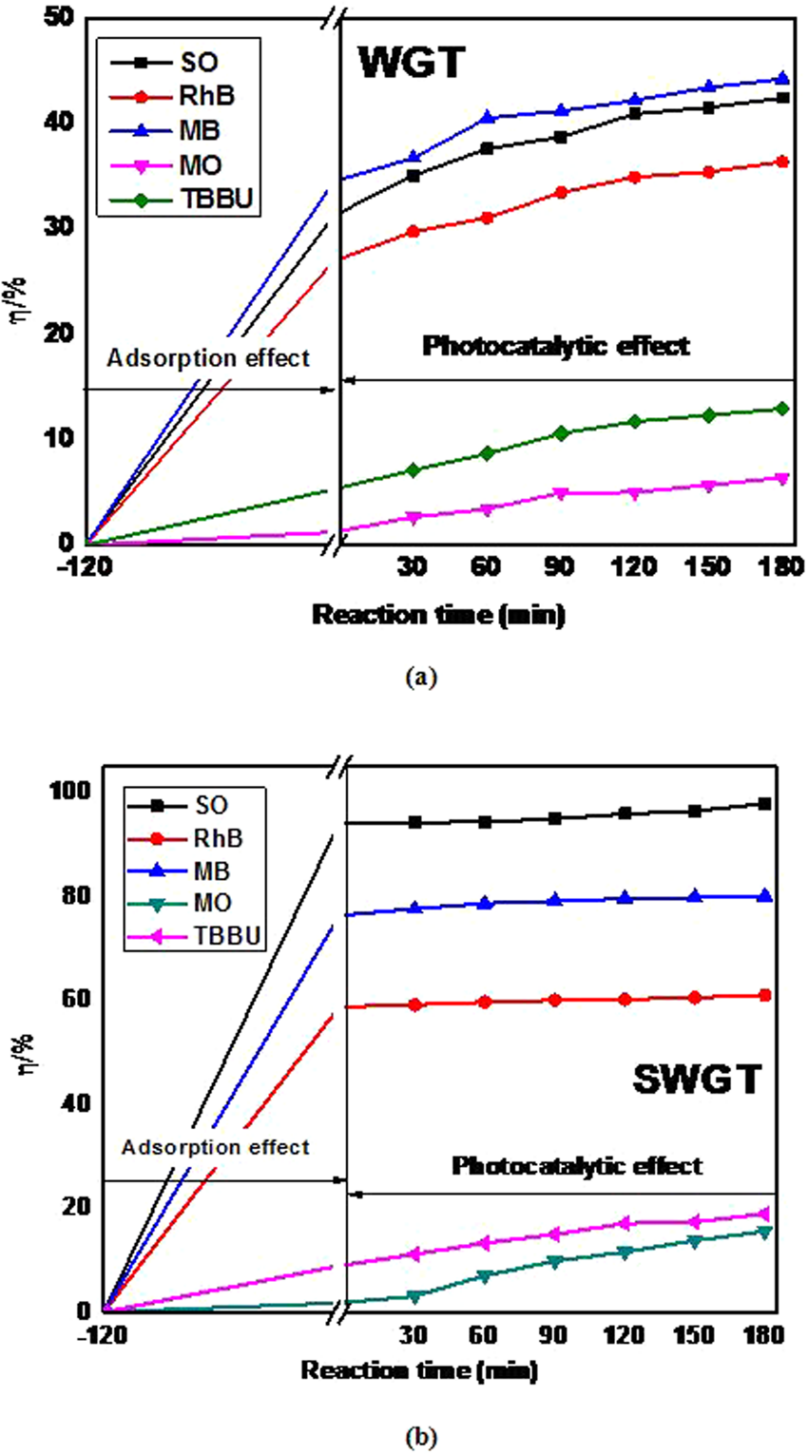
Degradation of WGT (**a**) and SWGT (**b**) composites for different dyes degradation under visible light irradiation. The amount of composite was 0.05 g. The experiments were carried out with neutral pH.

The photocatalytic activity over the SWGT composite was tested to evaluate the photocatalytic behavior of the SWGT composite at room temperature and typical atmospheric pressure for the degradation of SO, RhB, and MB as candidate sorbents from the cationic and anionic dye groups (MO, and TBBU) in aqueous solutions, as shown in Fig. 9.

Reviewing all photodegradation results in Fig. 9(a), the WGT nanocomposite exhibited good photodegradation results for degradation of MB organic dye solution. In the case of MB dye solution, the WGT composite has reached approximately 44.25% dye removal which is higher than that of SO and RhB was (42.43, and 36.39)%, respectively. In the case of anionic dyes, under presence of the WGT nanocomposite, degradation rate of TBBU organic dye revealed higher results than MO dye solution which reached approximately (12.95, and 6.42)% dye removal, respectively.

The photocatalytic degradation in Fig. 9(b) demonstrates that both the adsorption and the photodegradation effects in the presence of the SWGT composite were maximized and achieved the highest adsorption capability at about 94.19%. After 5 hours of the photocatalytic activity, the SWGT composite still demonstrated the best decolorization capability with 97.94% removal of the SO dye solution. In the case of cationic dye group, the SWGT composite also exhibited good decolorization capability for the RhB and MB solution cases with a final removal of organic dye at (61.03, and 80.06)%, respectively. On the contrary, the SWGT composite did not present good photocatalytic activity for the anionic dye group (MO, and TBBU). In the case of MO solutions, the SWGT composite exhibited low photocatalytic activity at 15.55%, which was lower than those of the cationic organic dyes. After 180 minutes under visible light irradiation, the SWGT composite displayed a photodegradation effect of 18.92% for the TBBU solution. Overall, the aforementioned results indicated that the SWGT composite is a good photocatalyst candidate for the degradation of cationic organic dyes with high photodegradation activity that far exceeds that of the anionic organic dyes.

**Figure 10:**
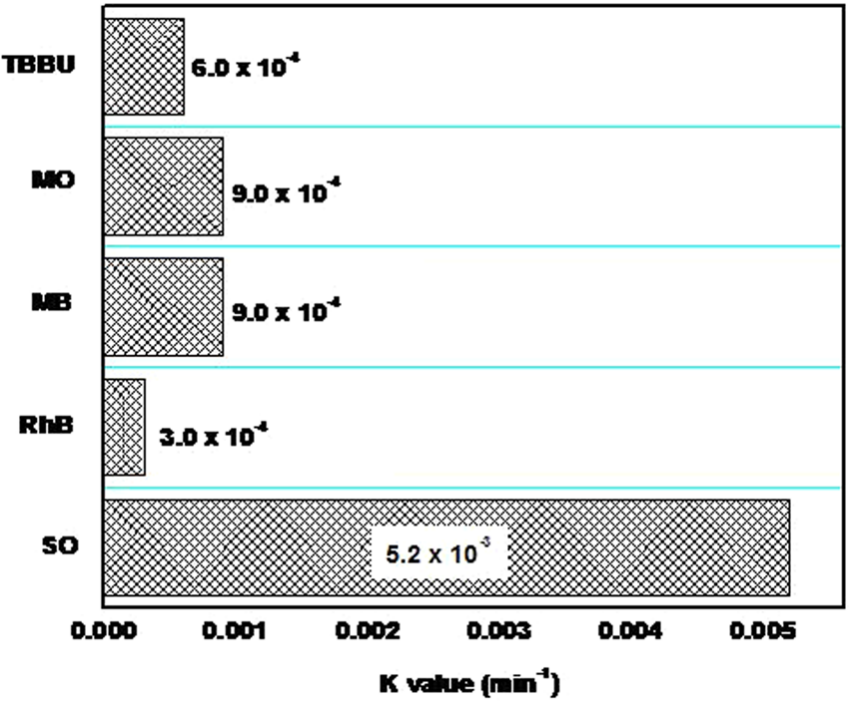
Kinetic plot for the degradation of SWGT composite for different dyes degradation under visible light irradiation. The amount of composite was 0.05 g. The experiments were carried out with neutral pH.

A plot of k_*app*_ is presented in Fig. 10 and Table 2. The SO degradation rate constant for the SWGT composite was 5.2 × 10^−3^ min^−1^, which was a better result than that of the other cationic-anionic organic dyes such as 3.0 × 10^−4^, 9.0 × 10^−4^, 9.0 × 10^−4^, and 6.0 × 10^−4^ min^−1^ corresponding to RhB, MB, MO, and TBBU, respectively. The SWGT composite is therefore a new potential material for photocatalyst activity.

**Table 2:** The apparent rate constant of SO, RhB, MB, MO and TBBU solutions by the SWGT nanocomposite.

K value (min^−1^)	SO	RhB	MB	MO	TBBU
SWGT	5.2 × 10^−3^	3.0 × 10^−4^	9.0 × 10^−4^	9.0 × 10^−4^	6.0 × 10^−4^

#### Survey of the effect of solution pH and dosage of catalyst

**Figure 11:**
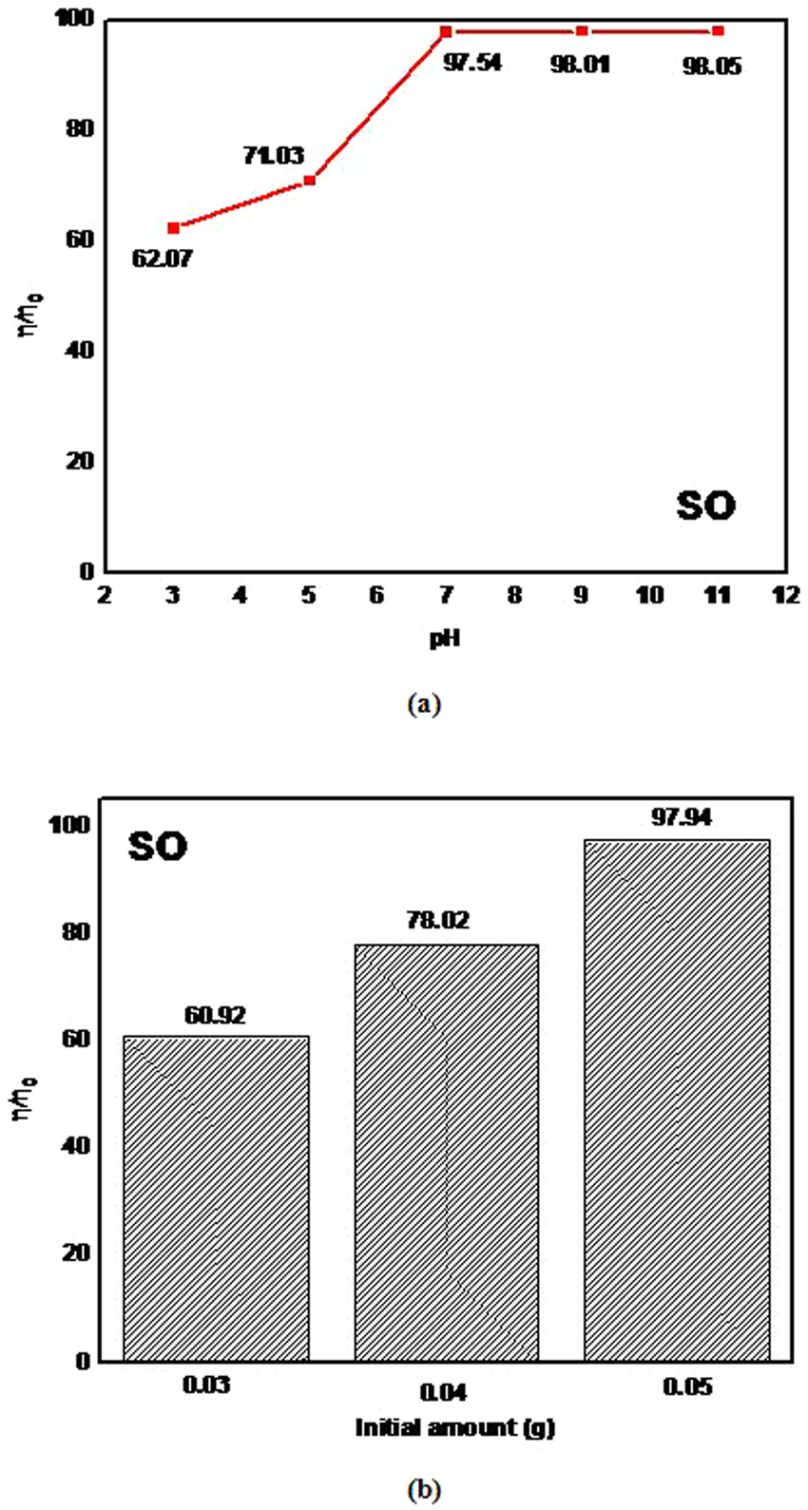
The effect of different solution pH (**a**) and the effect of different dosage amount (**b**) on the SWGT degradation of SO dye solution under visible light irradiation.

The SWGT composite retained the highest absorption capacity for the SO solution. This outcome was the reason for which we chose the SO solution to survey the effect of solution pH levels and the amount of composites in this study’s experiments. In the adsorption process, the pH factor exhibited an important role^3,60^. The pH value was tested, while keeping other parameters constant, at various initial pH levels between the range of 3–11 where the addition of the required amounts of 0.1 mol/L of NaOH or HCl solution was used to adjust the pH values. It clearly demonstrated the difference in the photocatalytic activity results of the pH solution in the presence of SWGT composite for the removal of SO organic dye. The photocatalytic activity results displayed in Fig. 11(a) demonstrate that the SO dye removal increased from 62.07% to 98.05% with the increase of the pH values from 3 to 11. The photodegradation effect was not more influenced in the pH ranges of 7 to 11; but, it far exceeded for the acid solution because the safranine O is a cationic (positive charge containing) dye^61^.

The photocatalytic degradation of the aqueous solution of SO was processed with different amounts that ranged between 0.03 to 0.05 g of the prepared SWGT composite in order to survey the effects of the initial amount of nanocomposite while keeping another parameters constant. With the decrease in the initial amount of catalyst, the degradation efficiency of the SO solution decreased. The decolorization capacity of the 0.05 g SWGT composite had the best result with 97.94% removal of dye after 5 hours for the photocatalytic activity. The photocatalytic activity results in Fig. 11(b) show that the dye removal decreased from 97.94% to 60.92% with the decrease in the dosage amount from 0.05 to 0.03 g in the SO solution. The interactive surface of the photocatalyst, as well as the fixed volume of the dye solution, decreased with the decreasing dosage amount of the photocatalyst; subsequently, the decolorization capacity of the composite decreased.

### Photocatalytic hydrogen production studies

**Figure 12:**
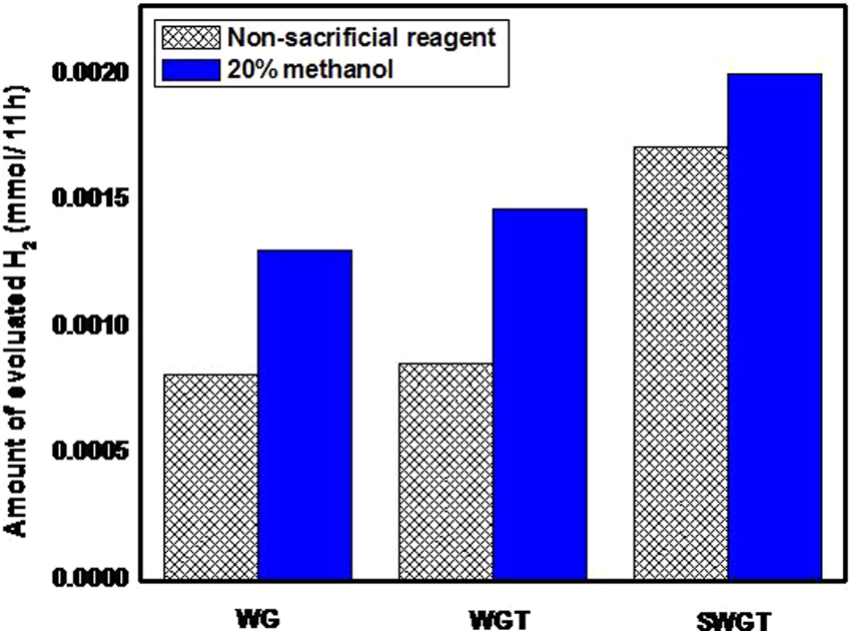
Hydrogen production rate from pure water and an aqueous solution containing 20% methanol with the SWGT composite as the photocatalyst.

For photocatalytic hydrogen evolution, the SWGT composite has a semiconductor role that converts sunlight energy into chemical energy under ambient conditions with and without sacrificial atmospheric pressure at room temperature^62–66^. In this experiment, methanol was used as the sacrificial reagent that further enhanced the catalytic activity of the semiconductor by providing electrons to consume the photogenerated holes which led the recombination time of the semiconductor to increase. The hydrogen evolution results for the SWGT composite with and without 20% methanol sacrificing reagents under visible light irradiation are provided in Fig. 12.

According to the hydrogen evolution results among the survey composites (Fig. 12**)**, the highest photocatalytic H_2_ evolution rate was observed when methanol was used as the sacrificial reagent in the presence of the SWGT composite. It achieved the H_2_ evolution rate of 2.004 mmol per 11 hours. However, the photocatalytic H_2_ evolution rate of the SWGT composite also reached a high value in pure water at 1.718 mmol per 11 hours. For the SWGT case, the presence of 20% methanol sacrificing reagents did not lead to a significant difference in the photocatalytic H_2_ evolution rates. It is a promising candidate for being a semiconductor in a highly active photocatalyst as well as obtaining great application and photocatalytic activity from a pure aqueous solution. Figure 12 displays the H_2_ evolution rate for WG and WGT photocatalysts. The H_2_ evolution rate of the WG composite from aqueous solution without the sacrificial reagent was 0.818 mmol per 11 hours; while it was 1.309 mmol per 11 hours in 20% methanol sacrificing reagents. About the WGT composite, the H_2_ evolution rate was 0.859 mmol per 11 hours and 1.472 mmol per 11 hours in the pure water and methanol aqueous solutions, respectively. According to the aforementioned results, the SWGT photocatalyst achieved the best hydrogen evolution rate, which is more than 2 times higher in both the pure water and methanol aqueous solutions. The above comparison implied a great photocatalytic hydrogen evolution rate than the ternary photocatalyst (WGT) and binary photocatalyst (WG).

## Conclusions

A SiO_2_/WSe_2_-graphene-TiO_2_ composite can be synthesized by using a simple self-assembly process. The main diffraction peaks of SiO_2_/WSe_2_-graphene-TiO_2_ composite was well identified by the SiO_2_, WSe_2_ and TiO_2_ signals that were investigated by XRD patterns. Nitrogen adsorption/desorption isotherms provided evidence that the SiO_2_/WSe_2_-graphene-TiO_2_ composite had not only the highest specific surface area but also exhibited a much stronger structure with the highest total pore volume than all other samples. Furthermore, the presence of the micro porous holes on the surface of the round SiO_2_ as well as WSe_2_ and TiO_2_ nanostructures were achieved through SEM and TEM imagery. The Raman, DRS and XPS data provided more information regarding the structure of the SiO_2_/WSe_2_-graphene-TiO_2_ composite.

The photodegradation experiments indicated that SiO_2_/WSe_2_-graphene-TiO_2_ composite is a good photocatalyst candidate for the degradation of cationic organic dyes with high photodegradation activity that far exceeds that of the anionic organic dyes. The optimal conditions for this study included a solution with a pH level of 11 and catalyst dosage 0.05 g for the SO solution case. The SiO_2_/WSe_2_-graphene-TiO_2_ photocatalyst achieved the best hydrogen evolution rate than the ternary photocatalyst (WGT) and binary photocatalyst (WG). The results of the characterization and the photodegradation suggested that SiO_2_/WSe_2_-graphene-TiO_2_ material is a promising material for the photodegradation of organic dyes as well as can facilitate the development of an efficient heterosystem for hydrogen production under visible light irradiation.

## Methods

### Reagents

Safranine O (SO, C_20_H_19_ClN_4_), texbrite BBU-L (TBBU), rhodamine B (RhB, C_28_H_31_ClN_2_O_3_), and methylene blue trihydrate (MB, C_18_H_18_ClN_3_S.3H_2_O) were purchased from the Samchun Pure Chemicals Co. Ltd., Korea. The tungsten (VI) oxide (WO_3_), selenium powder (Se, 99%), sodium sulfite (Na_2_SO_3_.7H_2_O, 95%), hydrochloric acid (HCl, 35.0–37.0%) and nitric acid (HNO_3_) were purchased from the Duskan Pure Chemicals Co. Ltd., Korea. Titanium (IV) oxide (TiO_2_, anatase, and nano power, 99.7%) was purchased from the Sigma-Aldrich Co. (USA). The tetraethyl orthosilicate (TEOS, 99%) that was used as the silica source was purchased from the Aldrich Chemistry, Germany. The cetyltrimethylammonium bromide (CTAB, C_19_H_42_BrN, 99%), methyl orange (MO, C_14_H_14_N_3_NaO_3_S), ammonium hydroxide (NH_4_OH), and ethylene glycol (C_2_H_6_O_2_, 99%) were purchased from the Daejung Chemicals Co. Ltd., Korea.

### Synthesis nanocomposites

#### Synthesis of the WSe_2_ nanocomposite

0.675 g tungsten (VI) oxide (WO_3_) was dissolved in 20 mL of distilled water, then dropped into 50 mL HNO_3_ 0.5 M in a three-necked flask (100 mL) and heated to 120 °C with magnetic stirring to eliminate the H_2_O and O_2_. Separately, a selenium salt was obtained by adding a combination of 0.01 mol anhydrous sodium sulfite (Na_2_SO_3_) and 0.004 mol crude selenium (Se) powder to 200 ml of ethylene glycol. A hydrothermal process at 180 °C for 36 hours was processed with both solutions. After washing step with 95% ethanol and distilled water, the solid was dried under a vacuum at 105 °C for 1 day to obtain the WSe_2_ material.

#### Synthesis of the WSe2 -graphene nanocomposite

Graphene oxide (0.2 g) in 100 ml of ethylene glycol was ultrasonicated for a half hour (Ultrasonic Processor, VCX 750, 500-Watt, Korea, Power 500-Watt, frequency 20KHz, Amplitude 50%, low intensity). The achieved WSe_2_ powder was mixed at equal volumetric ratios of 1:1 with strong stirring for 5 hours at 90 °C. A hydrothermal reaction at 100 °C for 10 hours was processed with the achieved solution. After washing step with 95% ethanol and distilled water, the WSe_2_-graphene nanocomposite were obtained after drying under a vacuum oven at 105 °C for 1 day and labeled as WG corresponding to the WSe_2_-graphene nanocomposite.

#### Synthesis of the WSe_2_–graphene-TiO_2_ nanocomposite

0.2 g graphene oxide was sonicated in 100 ml ethylene glycol for 30 minutes, followed by adding 0.1 g TiO_2_ nanopowder and 0.1 g the achieved WSe_2_ powder (0.1 g) with continued stirring for 5 hours at 90 °C. A hydrothermal reaction at 100 °C for 10 hours was processed with the achieved solution. After washing step with 95% ethanol and distilled water and drying under a vacuum oven at 105 °C for 1 day, the WSe_2_–graphene-TiO_2_ composite was synthesized. The sample was labeled as WGT corresponding to the WSe_2_-graphene-TiO_2_ nanocomposite.

#### Synthesis of the SiO_2_/WSe_2_–graphene-TiO_2_ nanocomposite

In general, part A was formed by the dissolution of 0.36 g CTAB in 57 mL of distilled water, then stirring for 30 min. By 30 min ultrasonicating of 0.4 g WGT in 20 ml of distilled water, part B was prepared. On the other beaker, the mixture of TEOS: ethanol: NH_3_ with volume ratio (ml) of 4:80:5 was combined to form part C. The mass ratio of SiO_2_/WSe_2_/graphene/TiO_2_ in the SWGT composite is fixed as 90:2.5:5:2.5. Then, part A, B, and C were then combined with strong stirring for 6 hours at an ambient temperature. After that, a certain amount of 25% NH_4_OH was added to the above dispersion until pH of 9.5–10 and then keep stirring for 1 hour. A hydrothermal reaction at 100 °C for 1 day was processed to obtain a final product. After washing step with 95% ethanol and distilled water and drying under a vacuum oven at 105 °C for 1 day, the obtained powder was kept in furnace to calcine at 25 °C to 550 °C for 8 hours, then it was heated at 550 °C for 6 hours under the ambient condition. The sample was labeled as SWGT corresponding to the SiO_2_/WSe_2_–graphene-TiO_2_ nanocomposite.

### Characterization

An X-ray diffraction was recorded using Shimadzu XD-D1. SEM was recorded using JSM-5600 JEOL, Japan. A DRS analysis was obtained by UV-vis spectrophotometry (Neosys-2000). TEM and selected-area electron diffraction (SAED) patterns were also used to investigate the size and distribution of the nanoparticles deposited on the graphene surface of the various samples. XPS analysis was observed using a VG Scientific ESCALAB250. Raman spectra can be obtained by a Jasco Model Name NRS-3100 spectrometry. Nitrogen adsorption/desorption isotherms studies were investigated by a Micromeritics ASAP 2020 M nvC operating at 77 K, the surface area was calculated by using the Brunauer-Emmett-Teller (BET) method. and the pore size distribution was calculated according to the Barrett-Joyner-Halenda (BJH) method. The photodegradation experiments were analyzed by a UV-spectrophotometry (Opizen POP, Korea). The photoluminescence (PL) spectra were recorded by a fluorescence spectrophotometer (F−4500, Hitachi, Japan) for an excitation wavelength of 325 nm at room temperature.

### Photocatalytic activity

The photodegradation experiment was processed under ambient conditions atmospheric pressure at room temperature without any sacrificial. Generally, 0.05 g SiO_2_/WSe_2_-graphene-TiO_2_ nanocomposite was dissolved in a 100-ml organic dye solution. The visible light source was made from an 8-watt lamp (Fawoo, Lumidas-H, Korea, λ ≥ 420 nm) with a filter (Kenko Zeta, transmittance m)90%). Firstly, a mixture solution of nanocomposite and organic dyes was kept without any light source for 120 min. The first sample was taken out at the end of 120 min kept in a dark box. The *c*_0_ is the concentration of dye solution at the starting point (*t* = 0). After that, other samples were taken out from the mixture solution each 30 min. Then, the powders were removed by using a centrifuge machine. The photocatalytic degradation of SO, RhB, MB, MO, and TBBU solutions tested after the above process with concentrations of 1 × 10^−4^, 5 × 10^−4^, 5 × 10^−4^, 5 × 10^−4^ and 1.25 × 10^−4^ mol/l, respectively.

On the other aspect, the effects of different initial pH levels (3–11) and catalyst dosages (0.03–0.05 g) were surveyed while keeping another parameter constant following by the photodegradation test. The effects of the above factors were expressed through the percent of dye removal. By using a UV-spectrophotometry, the concentration *c* the dye solutions can be obtained. The spectral range was surveyed at λ_max_ = 520, 554, 665, 465 and 349 nm for SO, RhB, MB, MO and TBBU, respectively. The degradation capacity (η%) was calculated as (1):

1 $$\eta \,( \% )=(1-c/{c}_{o})\times 100$$

### Photocatalytic hydrogen evolution system

Using the typical photocatalytic test conducted under ambient conditions with and without sacrificial atmospheric pressure, the SiO_2_/WSe_2_-graphene-TiO_2_ nanocomposite was 0.1 g, dissolved in a 200-ml solution. The solution of 20% methanol was used as a sacrificial reagent. The visible light source was made from an 8-watt lamp (Fawoo, Lumidas-H, Korea, λ ≥ 420 nm) with a filter (Kenko Zeta, transmittance >90%) to prevent any radiation below 410 nm and to ensure that the photocatalytic activity was conducted under visible light for 10 hours at 20 cm from the glass reactor. The amount of hydrogen gas evolved was measured at 25 °C with the atmospheric condition by a gas chromatograph (GC7900, Thermal conductivity detector), a molecular sieve 5A column. The nitrogen gas was used as the carrier gas.

## Data Availability

The datasets generated during and/or analyzed during the current study are available from the corresponding author on reasonable request.
